# The ASA Physical Status Classification: What Is the Evidence for Recommending Its Use in Veterinary Anesthesia?—A Systematic Review

**DOI:** 10.3389/fvets.2018.00204

**Published:** 2018-08-31

**Authors:** Karine Portier, Keila Kazue Ida

**Affiliations:** ^1^Univ Lyon, VetAgro Sup, GREAT, Marcy l'Etoile, France; ^2^Univ Lyon, CarMeN Laboratory, INSERM, INRA, INSA Lyon, Université Claude Bernard Lyon 1, Bron, France; ^3^Anesthésiologie et Réanimation Vétérinaires, Département de Clinique des Animaux de Compagnie et des Équidés, Université de Liège, Liège, Belgium

**Keywords:** mortality, fatal outcome, risk, dogs, cats, horses, rabbits, complications

## Abstract

**Background:** The effectiveness of the American Society of Anesthesiologists (ASA) Physical Status (PS) classification to identify the animals at a greater risk of anesthesia-related death and complications is controversial. In this systematic review, we aimed to analyze studies associating the ASA PS scores with the outcome of anesthesia and to verify whether there was any evidence for recommending the use of the ASA PS in veterinary patients.

**Methods:** Research articles found through a systematic literature search were assessed for eligibility, and data were extracted and analyzed using random-effects analysis.

**Results:** A total of 15 observational prospective and retrospective studies including 258,298 dogs, cats, rabbits, and pigs were included. The analysis found consistency between the studies showing that dogs, cats and rabbits with an ASA-PS ≥III had 3.26 times (95% CI = 3.04–3.49), 4.83 times (95% CI = 3.10–7.53), and 11.31 times (95% CI = 2.70–47.39), respectively, the risk of anesthesia-related death within 24 h (dogs) and 72 h (cats and rabbits) after anesthesia compared with those with an ASA PS <III. In addition, the analysis showed that dogs and cats with ASA PS ≥III had 2.34 times the risk of developing severe hypothermia during anesthesia (95% CI = 1.82–3.01).

**Conclusions:** The simple and practical ASA PS was shown to be a valuable prognostic tool and can be recommended to identify an increased risk of anesthetic mortality until 24–72 h after anesthesia, and a greater risk of development severe intraoperative hypothermia.

## Introduction

The American Society of Anesthesiologists (ASA) physical status (PS) consists of a classification system to assess a patient's physical status. The higher ASA PS appears to be related to a worse outcome of anesthesia. Its creation dates from 1941, when Saklad et al. were requested by the ASA to build a system that would allow retrieving statistical data in anesthesia (1). Their first task was to specify arbitrary definitions of numerous variables in order to establish standard terms and a common language. Initially, they intended to develop a tool to objectively assign an operative risk and establish a prognostic. However, in such approach, the statistical treatment was impossible due to the numerous variables associated with the different establishments and clinicians. They concluded that the term “operative risk” could not be used and it was more adequate to classify the patients according to their physical status only. They stated that “no attempt should be made to prognosticate the effect of a surgical procedure upon a patient of a given physical status,” since few variables were considered to favor the standardization of the definitions and the use of a common terminology for the statistical analysis.

**Figure 1:**
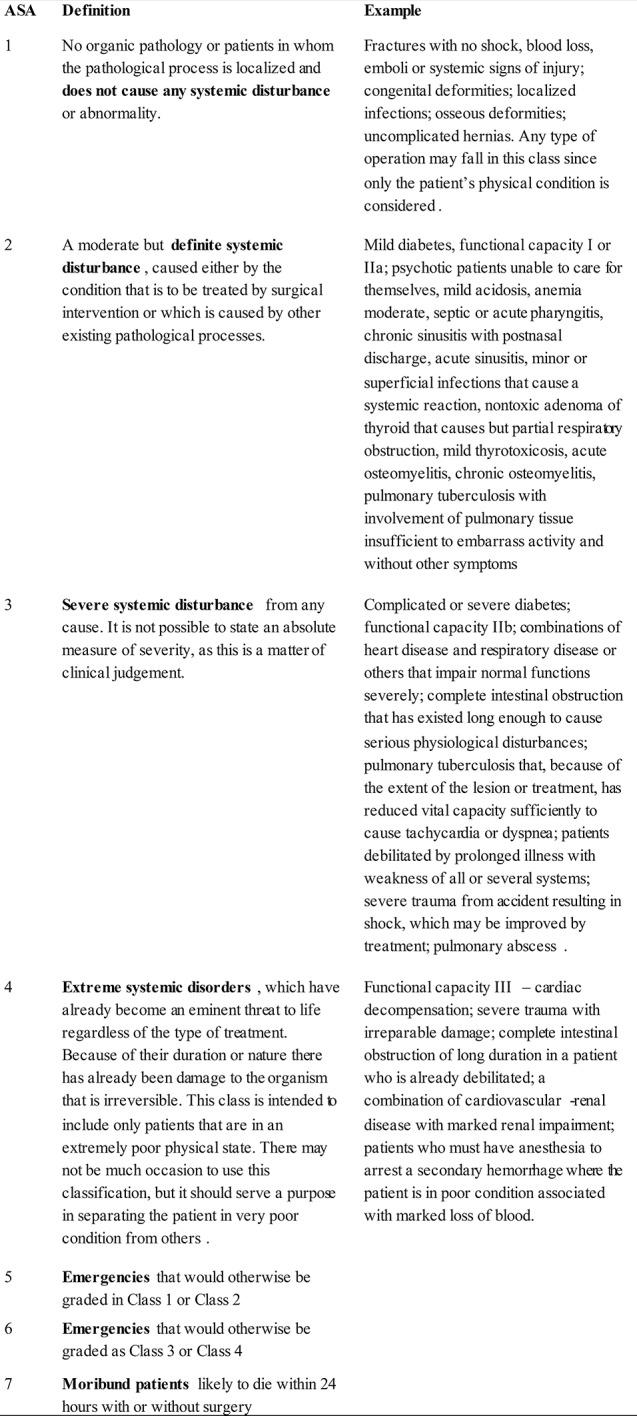
American Society of Anesthesiologists (ASA) grading of patients for surgical procedures according to Saklad (1).

At that time, there were different ways of assessing the patients' physical status, such as by assigning a number, a letter or, more explicit, a word (good, moderate, severe). An attempt to create a new method of standardization was proposed using six classes of “physical status” (Figure 1). The classes 1, 2, 3, and 4 consisted of systemic disturbances, which were graded into “none, definite, severe, extreme” with 5–10 examples each (1). The classes 5 and 6 consisted of the emergencies that would otherwise be graded in classes 1 or 2, and classes 3 or 4, respectively. A class 7 was added later to represent the moribund patients that were likely to die within 24 h with or without surgery.

The correlation between the incidence of mortality related to anesthesia and the physical status of the patient was shown for the first time in 1961 by Dripps et al. (2) in a study entitled “The role of anesthesia in surgical mortality.” In this study, the Arabic numbers from the classification of Saklad were modified to roman numbers, and the classes 5 and 6 were replaced by an “E” for “emergency” that could be added to each of the ASA classes. In addition, the grades “none, definitive, severe and extreme systemic disturbance” were replaced by “normal healthy, mild, severe, and incapacitating systemic disease” but these new definitions were not accompanied by examples. These modifications were accepted by the ASA in 1962 (3) and were published in the journal Anesthesiology in 1963 (4).

In 1978, the first study on the inter-anesthetists' variability concluded that the ASA PS classification was useful but was lacking scientific definition (5). Indeed, the terms used to define each class were subjective and inaccurate, and the qualitative adjectives, such as “mild, moderate, severe” implied a personal interpretation (6, 7). Additionally, the definitions based on the severity of the disease could also be controversial (8).

**Figure 2:**
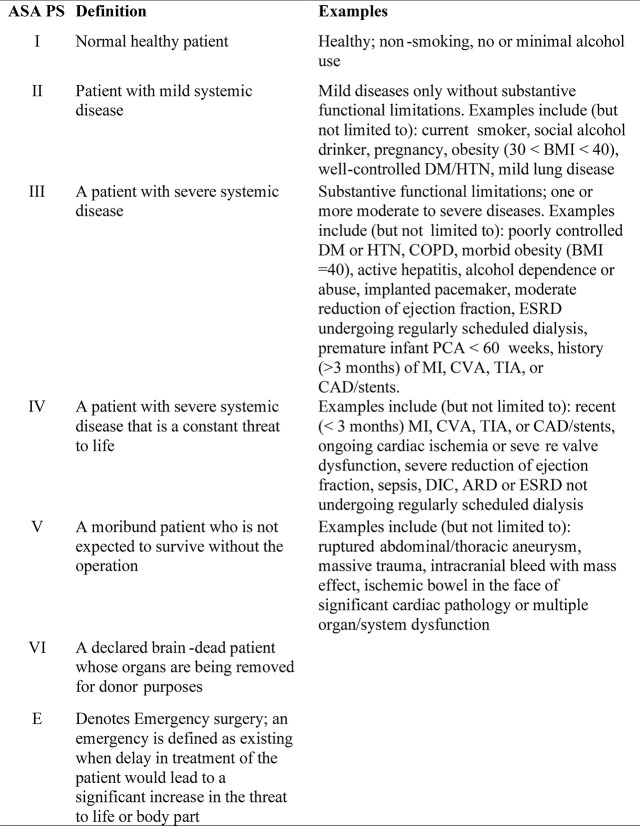
Current American Society of Anesthesiologists Physical Status (ASA PS) classification with definitions published in 1963 (4) and examples accepted in 2014 (9). BMI, body mass index; DM, diabetes mellitus; HTN, hypertension; COPD, chronic obstructive pulmonary disease; ESRD, end-stage renal disease; PCA, patient-controlled analgesia; MI, myocardial infarction; CVA, cerebrovascular accident; TIA, transient ischemic attack; CAD, coronary artery disease; DIC, disseminated intravascular coagulation; ARD, airway respiratory disease.

This subjectivity led to the last update of the classification system approved by the ASA House of Delegates (9) on October 15th 2014 (Figure 2). Most of the definitions were not modified, except for class V, in which the definition was changed from “a moribund patient who is not expected to survive for 24 h with or without surgery” to “a moribund patient who is not expected to survive for 24 h without operation.” Moreover, examples were added for each ASA PS class. For instance, smokers, alcoholics, pregnant women, and obese patients were included in classes II and III and an ASA VI category was added to include patients with brain-death and whose organs were being removed for donor purposes.

The actual version of the ASA PS classification was never validated in human medicine, although several studies showed the correlation between ASA PS and the risk of death (10–12) and complications associated with anesthesia. Such complications included the postoperative morbidity of patients after hip replacement surgeries, transurethral prostatectomy, cholecystectomy (13), and elective cranial neurosurgery (14); the incidence of infection, delayed wound healing, and deep vein thrombosis after plastic surgery (15) and; other major complications, such as atrial fibrillation, hypotension, and hypertension (16). In addition, high ASA PS scores were significantly correlated with long hospital and intensive care unit stays, high complication rates, and increased frequency of follow-ups (13). The ASA PS classification was equal to an index of physiological capacity to predict postoperative cardiovascular, respiratory, renal and infectious complications after major abdominal surgery (17). Intraoperative variables, such as duration of the surgery, duration of the assisted ventilation, and blood loss were also associated with the ASA PS score assigned preoperatively (18).

In veterinary medicine, to the authors' knowledge, one of the first prospective publications mentioning the association between the ASA PS classification and the anesthesia-related risk of death was from Clarke and Hall (19). Since then, several studies associating the ASA PS to anesthesia-related risk of death were published for dogs and cats (20–34), rabbits (24, 35), pigs (36), and horses (37, 38) with different outcomes and definitions. However, whether veterinary patients with a high ASA PS score are at an increased risk of death and development of complications associated with anesthesia remains unknown.

In this systematic review, we compared the studies assessing the ASA PS with the outcome of anesthesia in domestic animals, aiming to verify whether there was evidence that the ASA PS was actually effective to identify patients at a higher risk of anesthesia-related death or at a higher risk of developing any complication associated with anesthesia.

## Methods

### Online database search strategy

In order to find the studies assessing the anesthesia-related death and complications, an online database search was performed. In the online search, the terms (ASA or American-Society-of-Anesthesiologists) and (anesthesia or anaesthesia) and (death or mortality or risk or morbidity or complication or outcome) and (veterinary or animal) were entered in Pubmed, Google Scholar, Scopus, and VetMed Resources on April 1st 2018. In VetMed Resource, the results were filtered by “journal article,” “English language,” “death rate,” ”morbidity,” and “clinical aspects.” One paper was hand searched from the reference section of other papers and books.

The outcome variables included anesthesia-related mortality and complications in any domestic animal species. The anesthesia-related mortality was defined as death where anesthesia could not be excluded as a potential cause. The anesthesia-related complications were defined as any clinical alteration where anesthesia could not be excluded as a potential cause.

Only published research articles in peer-reviewed journals providing the outcome (which could be death or any other complication associated with anesthesia) according to the ASA PS score were included in the study. Studies in any domestic animal species or specific study population of domestic animals were considered for inclusion. The studies were grouped by outcome, i.e., mortality and complications, and then by animal species and specific group populations. The patients were assessed according to their ASA PS scores, which could be ASA PS <III, defined as healthy patients or with mild diseases only, without substantive functional limitations, or ASA PS ≥III, defined as sick patients with one or more moderate to severe diseases and substantive functional limitations (4, 9). The division of the ASA PS scores into two groups aimed to facilitate the analysis and was based in previous large studies assessing anesthesia-related mortality in veterinary patients (19, 24).

### Risk of bias assessment

**Figure 3:**
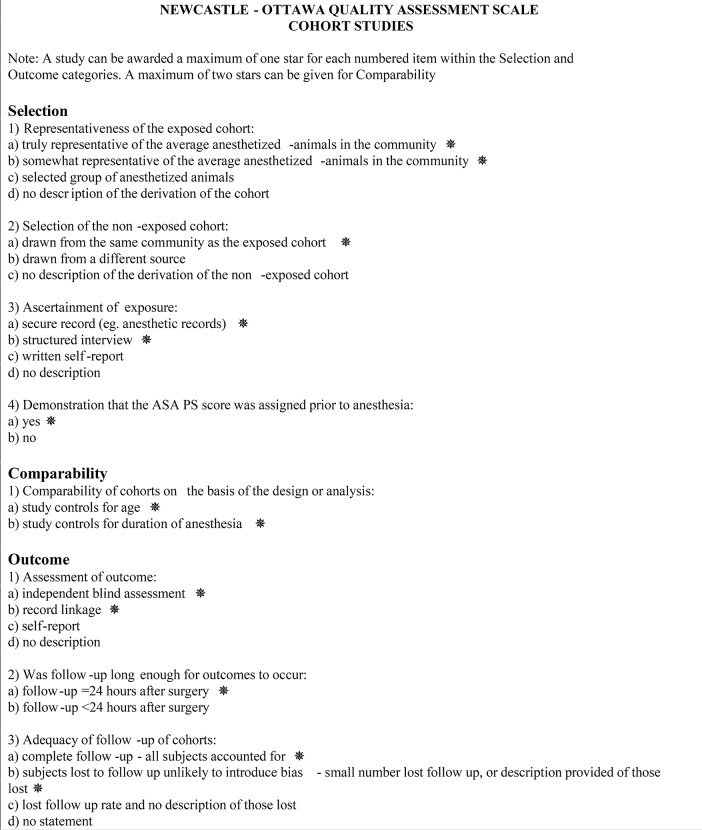
Newcastle-Ottawa scale for assessment of quality of non-randomized studies included in the analysis.

The risk of bias was evaluated for each article using a 9-point Newcastle-Ottawa scale (Figure 3) to assess the quality of non-randomized studies included in systematic reviews and meta-analyses (39). In this scale, each study was assigned a maximum of 4 points for quality of selection, 2 points for comparability, and 3 points for quality of outcome and adequacy of follow-up. The sum of the points from each category consisted of the Newcastle-Ottawa score, which indicated a low, moderate, and high risk of bias for 7–9, 4–6, and 1–3 points, respectively (40).

### Study heterogeneity

To verify the consistency of the findings of the studies assessing the same outcome in the same animal species, the Cochrane's *Q* and the *I*^2^ heterogeneity tests were calculated. The Cochran's *Q* indicated whether the variations between the results were genuine (*P* < 0.05 = heterogeneity) or attributable to chance (*P* > 0.05 = homogeneity). The proportion of the inconsistency (heterogeneity) was expressed by the *I*^2^ statistic between 0 and 100% [*I*^2^ = 100% x (Cochran's Q–degree of freedom)/Cochran's Q]. Negative values for *I*^2^ were considered equal to 0% (41).

### Statistical analyses

**Figure 4:**
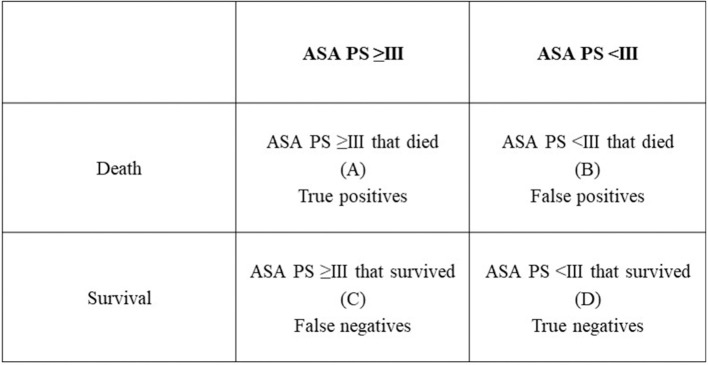
The 2 × 2 table for binary outcomes used for assessing the relative risk and the 95% confidence interval in the present study.

A 2 × 2 table for binary outcomes (Figure 4) was extracted from each study. From this table, the relative risk (RR) and the 95% confidence interval (CI) were calculated for each study according to the following equation: RR = [A/(A+B)]/[C/(C+D)]. The experimental group was defined as patients ASA PS ≥III and the control group was defined as patients ASA PS <III. A RR < 1.0 (plotted to the left of the line 1.0 in the graphs) indicated that in that study, patients with ASA PS ≥III were at a lower risk of anesthesia-related morbidity or mortality compared with ASA PS <III. A RR > 1.0 (plotted to the right of the line 1.0 in the graphs) indicated that in that study, patients with ASA PS ≥III were at a higher risk of anesthesia-related morbidity or mortality compared to patients with ASA PS <III. A RR = 1.0 indicated there was no difference in risk of anesthesia-related morbidity or mortality for patients assigned either ASA PS <III or ASA PS ≥III. The random-effects statistical model, which allows for differences in the treatment effect from study to study, was used for this analysis (42). The RR, the Cochran's *Q*, and the *I*^2^ were calculated using MedCalc Statistical Software version 18.2.1 (MedCalc Software bvba, Ostend, Belgium).

## Results

### Studies included in the analysis

**Figure 5:**
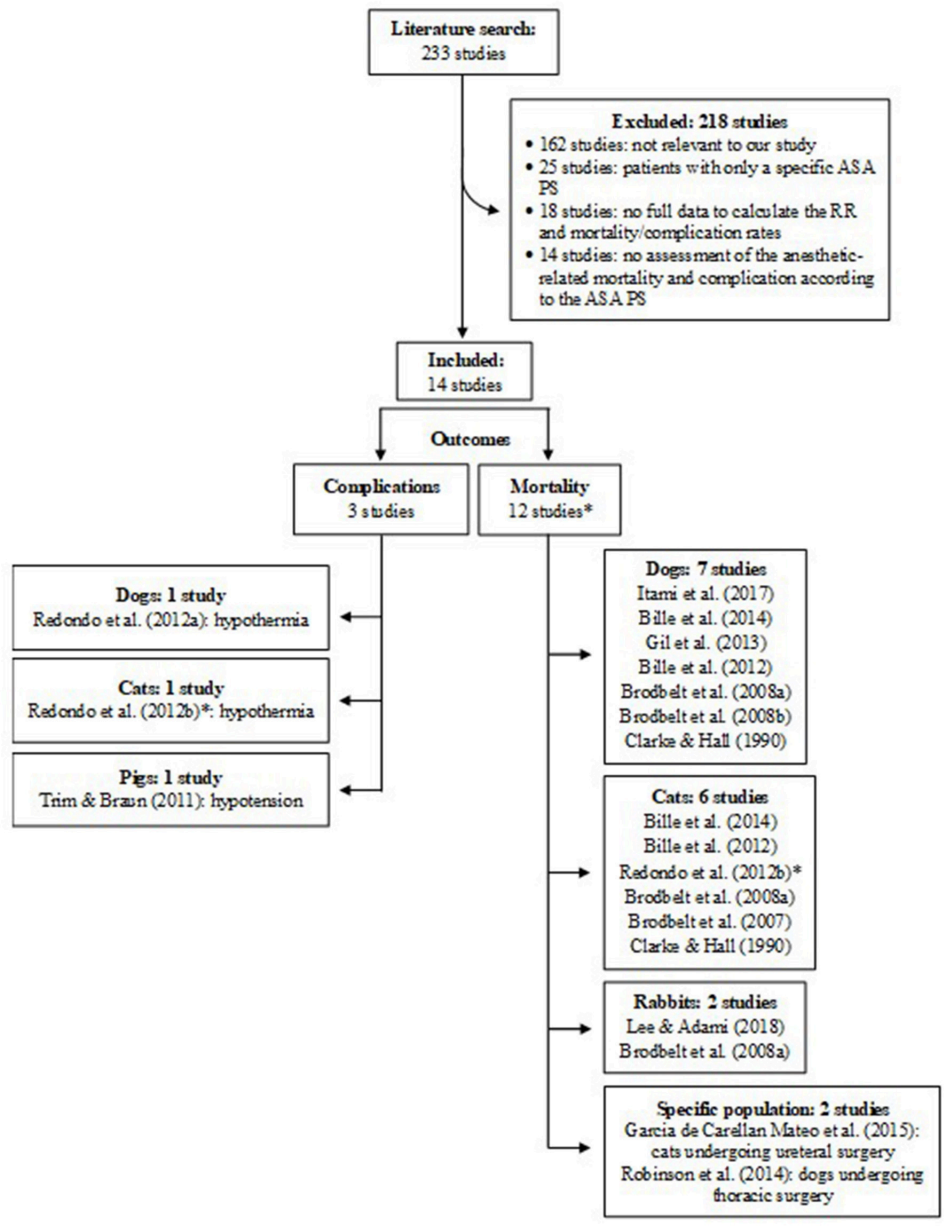
Flow diagram of studies included in the analysis. *The study of Redondo et al. (28) was counted twice because it assessed both complication and mortality. RR, relative risk; ASA PS, American Society of Anesthesiologists physical status.

A total of 233 studies were retrieved using the online research database strategy (65 from Pubmed, 14 from Google Scholar, 5 from Scopus, and 148 from VetMed Resources) and by hand searching the literature (1 study). From these, 162 were excluded because, based on the abstract, they were not relevant to our study, 25 studies were excluded because of the inclusion of patients with only a specific ASA PS, 18 were excluded because of no full data provision to calculate the RR and incidence of mortality or complication, and 14 studies were excluded because of no assessment of the anesthetic-related mortality and complication according to the ASA PS (Figure 5).

A total of 14 studies with 241,509 patients (131,024 dogs; 102,064 cats; 8,394 rabbits; and 27 pigs) from 236 clinics (1 from USA, 1 from France, 18 from Japan, 42 from Spain, and 174 from UK) assessed from 1984 to 2016 met the inclusion criteria (Table 1). Studies in other animal species, such as horses and birds, did not comply with the inclusion criteria.

**Table 1:** Study design and population, number of patients included in the study, overall mortality, period of the study, number of clinics, and country of the studies included in the review.

**Studies**	**Study design**	**Population**	***n* included (overall mortality)**	**Period of the study**	***n* clinics**	**Country**
**MORTALITY IN DOGS**						
Itami et al. (33)	Observational prospective cohort	Dogs anesthetized for surgical or diagnostic procedures	4,323 (0.65%)	Apr 2010–Mar 2011	18	Japan
Bille et al. (30)	Observational prospective cohort	Dogs undergoing general anesthesia	1,783 (0.62%)	Apr 2008–Apr 2010	1	France
Gil and Redondo (29)	Observational prospective cohort	Dogs undergoing anesthesia	2,012 (1.29%)	Feb 2007–Mar 2008	39	Spain
Bille et al. (26)	Observational prospective cohort	Dogs and cats undergoing general anesthesia	2,252 (1.51%)	Apr 2008–Apr 2010	1	France
Brodbelt et al. (24, 25)	Observational prospective cohort	Dog undergoing anesthesia and sedation	98,036 (0.17%)^a^	Jun 2002–Jun 2004	117	UK
Clarke and Hall (19)	Observational prospective cohort	Dogs undergoing anesthesia	20,814^a^ (0.23%)	1984–1986	53	UK
**MORTALITY IN CATS**						
Bille et al. (30)	Observational prospective cohort	Dogs undergoing general anesthesia	902 (1.11%)	Apr 2008–Apr 2010	1	France
Bille et al. (26)	Observational prospective cohort	Dogs undergoing general anesthesia	1,294 (1.08%)	Apr 2008–Apr 2010	1	France
Redondo et al. (28)	Retrospective	Cats undergoing anesthesia	275 (2.2%)	Not available	1	UK
Brodbelt et al. (23, 24)	Observational prospective cohort	Cats undergoing anesthesia and sedation	79,178 (0.24%)^a^	Jun 2002–Jun 2004	117	UK
Clarke and Hall (19)	Observational prospective cohort	Cats undergoing anesthesia	20,103 (0.29%)^a^	1984–1986	53	UK
**MORTALITY IN RABBITS**						
Lee et al. (35)	Retrospective	Anesthetized and sedated pet rabbits	185 (18.5%)^a^	2009–2016	1	UK
Brodbelt et al. (24)	Observational prospective cohort	Rabbits undergoing anesthesia and sedation	8,209 (1.39%)^a^	Jun 2002–Jun 2004	117	UK
**MORTALITY IN SPECIFIC POPULATION**						
Garcia de Carellan Mateo et al. (32)	Retrospective cohort	Cats anesthetized for ureteral surgery	37 (18.9%)	Mar 2010–Mar 2013	1	UK
Robinson et al. (31)	Retrospective	Dogs undergoing thoracic surgery	279 (2.2%)—at 24 h 266 (3.6%)—at discharge	Jun 2002–Jun 2011	1	UK
**COMPLICATIONS**						
Redondo et al. (27)	Retrospective	Dogs undergoing anesthesia	1,525	Not available	2	Spain
Redondo et al. (28)	Retrospective	Cats undergoing anesthesia	275	Not available	1	Spain
Trim and Braun (36)	Retrospective	Pigs undergoing anesthesia	27	May 1999–Jun 2006	1	USA

a *Including euthanized patients*.

There were 12 studies assessing mortality and 3 studies assessing complications included in the analysis (1 study assessed both mortality and complications) (Figure 5). Mortality was assessed according to the animal species (7 studies in dogs, 6 studies in cats, 2 studies in rabbits), and according to specific populations (i.e., dogs undergoing thoracic surgery, cats undergoing ureteral surgery). Complications included the development of hypothermia, hyperthermia, and hypotension.

All studies had a low risk of bias, except the ones of Clarke and Hall (19) and Lee et al. (35), which had a moderate risk of bias. The study of Clarke and Hall (19) mentioned that animals died during or shortly after surgery but they did not specify the exact length of follow-up. Lee et al. (35) assigned the ASA PS score retrospectively from the animal records.

All studies excluded animals that died due to euthanasia from the analysis, except for the studies of Clarke and Hall (19), Brodbelt et al. (24), and Lee et al. (35).

The studies from Brodbelt et al. (23, 25) had supplementary data of the study of Brodbelt et al. (24) and were included in the analysis only to assess the risk of bias of the latter.

### Anesthesia-related mortality in dogs

**Figure 6:**
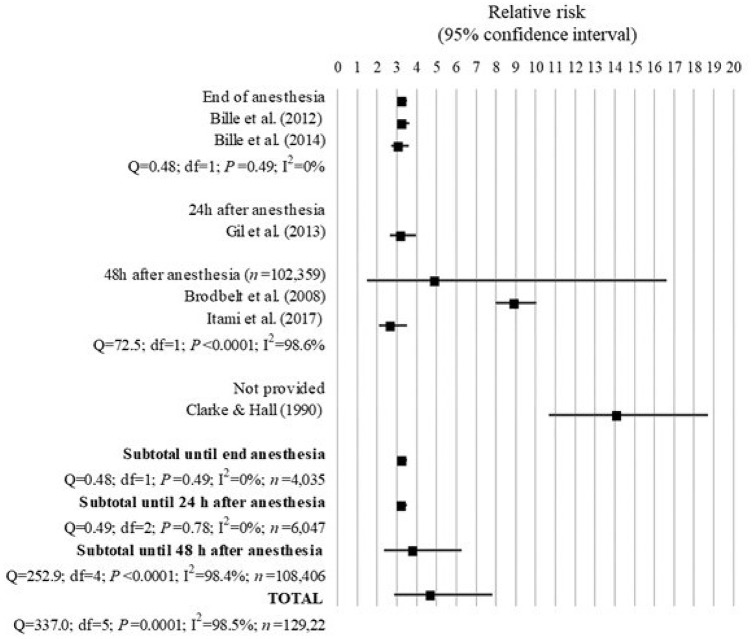
Forest plot showing the increased risk of anesthesia-related death in dogs with ASA PS ≥III compared with ASA PS < III. *Q*, Cochran's *Q* (*P* < 0.05 = heterogeneity; *P* > 0.05 = homogeneity); *I*^2^, proportion of the inconsistency between the findings of the studies; *df* , degrees of freedom.

Six studies assessing the anesthesia-related death in dogs were included in the analysis (Table 1 and Figure 6). All studies, except for Brodbelt et al. (24), excluded euthanized dogs from the analysis because deaths were not associated with anesthesia.

The overall mortality rate associated with anesthesia shown in the studies analyzed decreased from 0.23 to 0.17% between 1976–1978 and 2002–2004 (19, 24). This was mainly because of a decrease in the mortality rate of ASA PS III-V from 3.12 to 1.33%, although the proportion of deaths in ASA PS I-II also decreased from 0.11 to 0.05%.

All studies found a significant greater risk of anesthesia-related death in dogs with ASA PS ≥III compared to dogs with ASA PS <III. Overall the combined results of the studies showed that dogs with ASA PS ≥III had 4.73 times the risk of death due to causes associated with anesthesia compared to dogs with ASA PS <III (95% IC = 2.87 to 7.81; *P* < 0.001). However, there was a significant inconsistency of 98.5% (*Q* = 337.0; *P* < 0.0001; *I*^2^ = 98.5%) between the findings of all studies, which was further investigated by analyzing the studies according to their length of follow-up.

Further investigation revealed 0% heterogeneity between the studies of Bille et al. (26) and Bille et al. (30), which assessed death until the end of anesthesia (*Q* = 0.48; *df* = 1; *P* = 0.49), and between these studies and the study of Gil and Redondo (29), which assessed death until 24 h after anesthesia (*Q* = 0.49; *df* = 2; *P* = 0.78). They found that dogs with ASA PS ≥III had 3.26 times the risk of anesthesia-related death until the end of anesthesia (95% CI = 3.03 to 3.51; *P* < 0.001) and until 24 h after anesthesia (95% CI = 3.04 to 3.45; *P* < 0.001) compared to dogs with ASA PS <III.

When prolonging the length of follow-up to 48 h after anesthesia, the studies of Brodbelt et al. (24) and Itami et al. (33) found that dogs ASA PS ≥III had 8.95 times (95% IC = 7.97–10.04; *P* < 0.0001) and 2.71 times (95% IC = 2.09–3.51; *P* < 0.0001) the risk of anesthesia-related death, respectively, although there was 98.6% inconsistency (*Q* = 72.5; *df* = 1; *P* < 0.0001) between the findings of these studies.

The study of Clarke and Hall (19) found the highest risk of 14.14 times for anesthesia-associated death in dogs with ASA PS ≥III compared to dogs with ASA PS <III (95% CI = 10.68 to 18.71; *P* < 0.0001), although the length of follow-up was not provided in the article.

### Anesthesia-related mortality in cats

**Figure 7:**
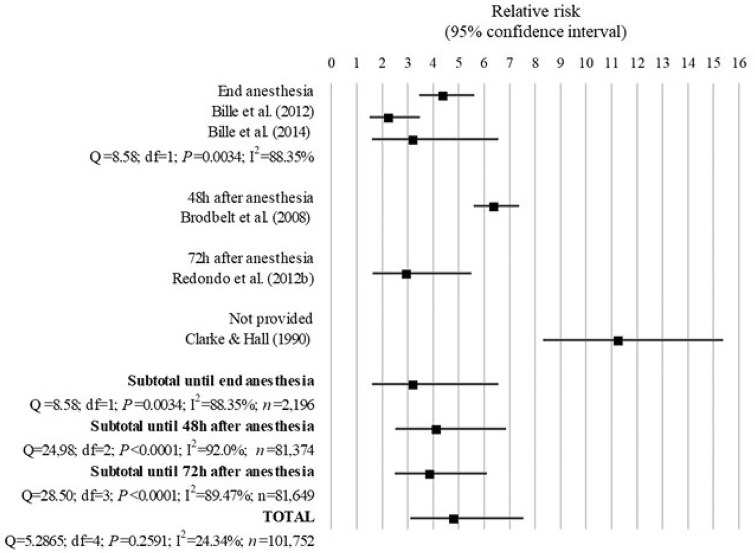
Forest plot showing the increased risk of anesthesia-related death in cats with ASA PS ≥III compared with ASA PS <III. *Q*, Cochran's *Q* (*P* < 0.05 = heterogeneity; *P* > 0.05 = homogeneity); *I*^2^, proportion of the inconsistency between the findings of the studies; *df* , degrees of freedom.

The 5 studies assessing the anesthesia-related mortality on cats included in the analysis were presented in Table 1 and Figure 7.

The overall mortality rate associated with anesthesia shown in the studies analyzed decreased from 0.29% (19) to 0.24% (24) between 1976–1978 and 2002–2004, mainly because of a decrease in the mortality rate of ASA PS III-V from 3.33 to 1.4%, although the proportion of deaths in ASA PS I-II also decreased from 0.18 to 0.11%. Animals dying due to euthanasia were excluded from the analysis on 3 (26, 28, 30) out of 5 studies in cats.

All studies showed a significant greater risk of anesthesia-related death in cats with ASA PS ≥III compared with ASA PS <III. The studies of Bille et al. (26) and Bille et al. (30) found that cats with ASA PS ≥III had 3.24 times (95% CI = 1.60 to 6.55; *P* = 0.001) the risk of anesthesia-related death until the end of anesthesia than cats ASA PS <III, although there was a significant 88.35% inconsistency between these results (*Q* = 8.58; *df* = 1; *P* = 0.0034). The studies of Brodbelt et al. (24) and Redondo et al. (28) found that cats ASA PS ≥III had 6.42 times (95% CI = 5.58–7.38; *P* < 0.0001) and 2.99 times (95% CI = 1.63–5.49; *P* = 0.0004) the risk of anesthesia-associated death until 48 and 72 h after anesthesia, respectively, compared to cats with ASA PS <III, although a significant heterogeneity was found between the results of these studies (*Q* = 72.5; *df* = 1; *P* < 0.0001; *I*^2^ = 98.6%). Clarke and Hall (19) found that cats ASA PS ≥III had 11.3 times (95% = CI 8.31–15.3; *P* < 0.0001) the risk of death due to causes associated with anesthesia compared to cats with ASA PS <III, although no length of follow-up was provided in the study.

The overall RR for the 5 studies combined showed that cats with ASA PS ≥III had 4.83 times (95% CI = 3.10–7.53; *P* < 0.001) greater risk of anesthesia-related mortality compared to cats with ASA-PS <III. No significant inconsistency was detected between the results of these studies (*I*^2^ = 24.34%; *Q* = 5.2865; *df* 4; *P* = 0.2591; Figure 7).

### Anesthesia-related mortality in rabbits

**Figure 8:**
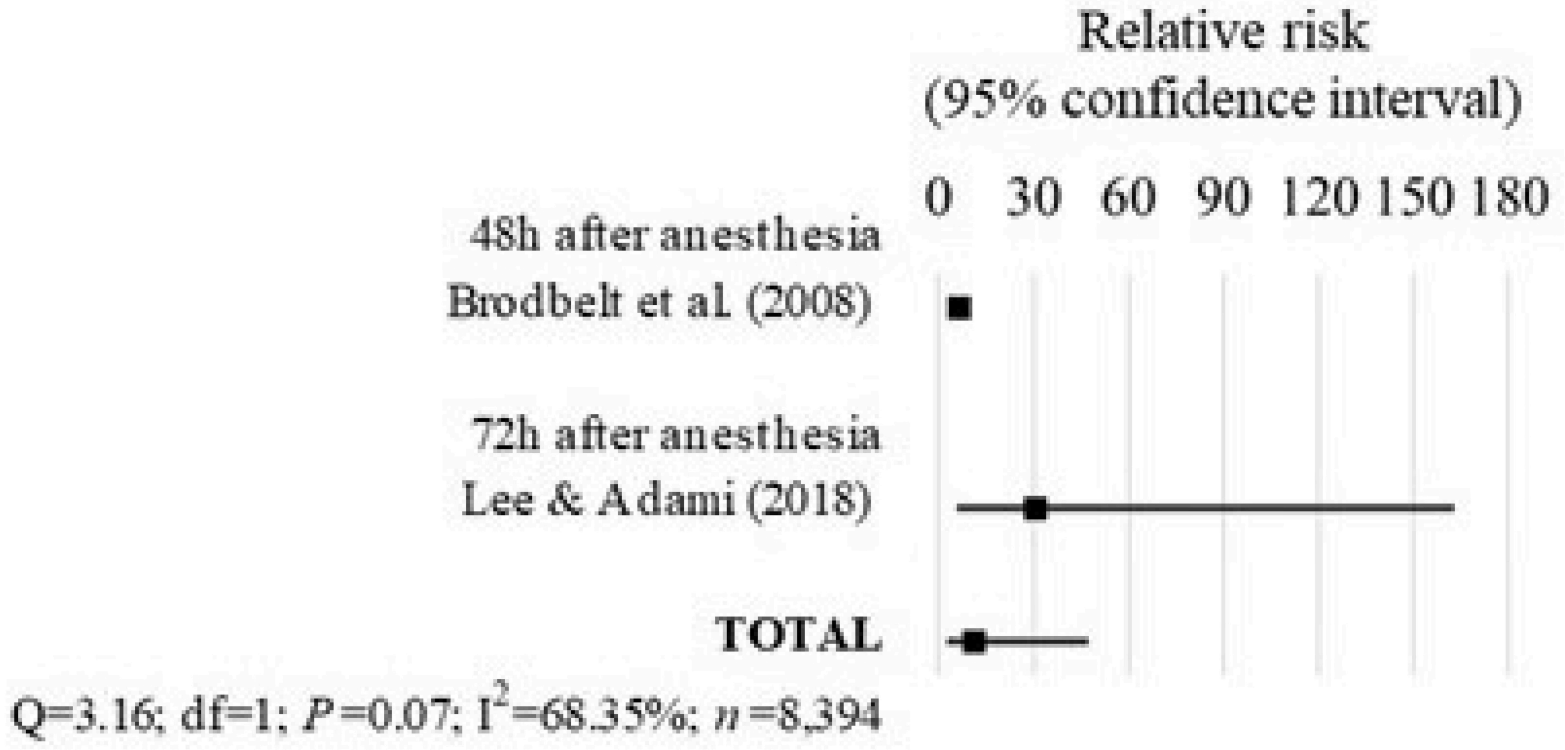
Forest plot showing the increased risk of anesthesia-related death in rabbits with ASA PS ≥III compared with ASA PS <III. *Q*, Cochran's *Q* (*P* < 0.05 = heterogeneity; *P* > 0.05 = homogeneity); *I*^2^, proportion of the inconsistency between the findings of the studies; *df* , degrees of freedom.

Two studies assessing the mortality associated with anesthesia on rabbits were included in the analysis (Table 1 and Figure 8).

In the study of Brodbelt et al. (24), the rabbits with ASA PS ≥III had 6.64 times the risk of anesthesia-related death until 48 h after anesthesia compared to rabbits with ASA PS <III (95% CI = 5.19–8.51; *P* < 0.0001).

The study of Lee et al. (35) found that rabbits with ASA-PS ≥III had 30.6 times the risk of anesthesia-associated death until 72 h after anesthesia compared to rabbits with ASA PS <III (95% CI = 5.74–163.1; *P* = 0.0001). In this study, the ASA PS scores were assigned retrospectively from the patient's records, which contributed to a moderate risk of bias.

Overall, the findings of these studies combined showed that rabbits with ASA PS ≥III had 11.31 times the risk of death-associated with anesthesia compared to rabbits with ASA PS <III (95% CI = 2.70–47.39; *P* = 0.001). There was no significant heterogeneity between the findings of these studies (*Q* = 3.16; *df* = 1; *P* = 0.07; *I*^2^ = 68.35%), regardless of the differences in the length of follow-up of 48 h (24) and 72 h after anesthesia (35).

### Anesthesia-related mortality in specific populations

**Figure 9:**
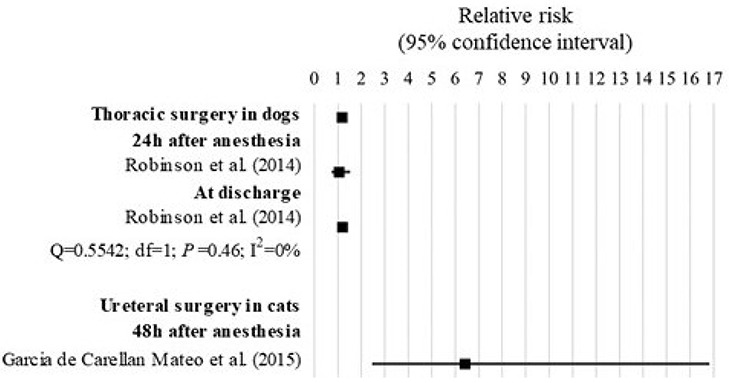
Forest plot showing the increased risk of anesthesia-related death in specific populations with ASA PS ≥III compared with ASA PS <III. *Q*, Cochran's *Q* (*P* < 0.05 = heterogeneity; *P* > 0.05 = homogeneity); *I*^2^, proportion of the inconsistency between the findings of the studies; *df* , degrees of freedom.

There were 2 studies included in the analysis that assessed the risk of death on specific populations, which were dogs undergoing thoracic surgery and cats undergoing ureteral surgery (Table 1 and Figure 9).

In the study of Robinson et al. (31), dogs ASA PS ≥III undergoing thoracic surgery had 1.19 times the risk of anesthesia-related death compared to those with ASA PS <III (95% CI = 1.04 to 1.36; *P* = 0.01). Within the study, although the risk of death was significant when assessed until discharge (RR = 1.21; 85% CI = 1.05–1.40; *P* = 0.0079) but not when assessed until 24 h after anesthesia (RR = 1.06; 95% CI = 0.74–1.52; *P* = 0.7603), no significant heterogeneity was found between the findings of the study (*Q* = 0.55; *df* = 1; *P* = 0.46; *I*^2^ = 0%).

In the study of Garcia de Carellan Mateo et al. (32), cats ASA PS ≥III undergoing ureteral surgery had 16.43 times the risk of anesthesia-related mortality compared to cats with ASA PS <III (95% CI = 2.46–16.81; *P* = 0.0001).

## Complications

**Figure 10:**
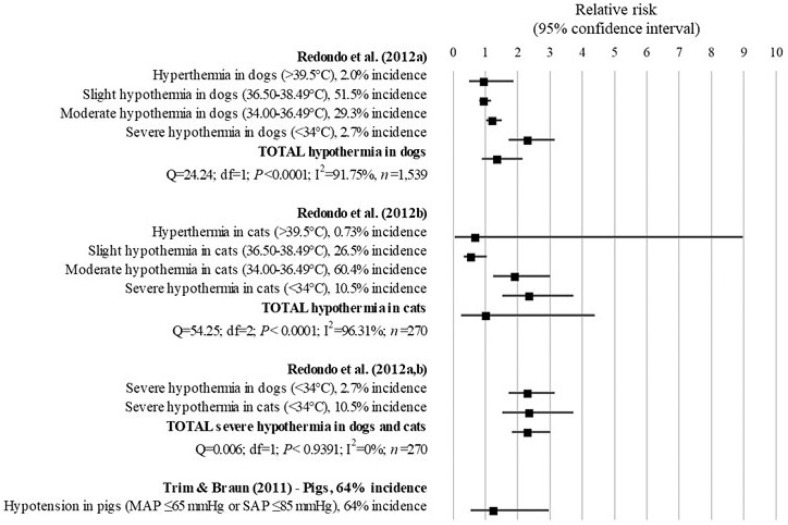
Forest plot showing the increased risk of developing complications associated with anesthesia in patients with ASA PS ≥III compared with ASA PS <III. *Q*, Cochran's *Q* (*P* < 0.05 = heterogeneity; *P* > 0.05 = homogeneity); *I*^2^, proportion of the inconsistency between the findings of the studies; *df* , degrees of freedom.

Three studies describing anesthesia-related complications were included in the analysis (Figure 10). All studies used a retrospective design. The complications consisted of: hypothermia, which was stratified in three levels [i.e., slight (36.5–38.49°C), moderate (34.0–36.49°C), and severe (< 34°C)], and hyperthermia (>39.5°C) in dogs and cats at the end of anesthesia; and arterial hypotension (MAP ≤ 65 mmHg or SAP ≤ 85 mmHg) in Vietnamese potbellied pigs at discharge.

In dogs, the study of Redondo et al. (27) found that the risk of developing hyperthermia (RR = 1.00; 95% CI = 0.50–1.87; *P* = 0.9195) and hypothermia (RR = 1.39; 95% CI = 0.90–2.15; *P* = 0.14) was not significantly different between patients with ASA PS ≥III compared to patients with ASA PS <III. However, when stratifying hypothermia in three levels, dogs with ASA PS ≥III had 1.24 times (95% CI = 1.03–1.50; *P* = 0.0252) and 2.33 times (95% CI = 1.72–3.15; *P* < 0.0001) the risk of developing moderate and severe hypothermia associated with anesthesia, respectively, compared to dogs with ASA PS <III.

In cats, similar to dogs, the study of Redondo et al. (28) showed no significant difference in the risk for developing hyperthermia (RR = 0.71; 95% CI = 0.06–8.97) and hypothermia (RR = 1.04; 95% CI = 0.25–4.38; *P* = 0.95) between cats with ASA PS ≥III compared to cats with ASA PS <III. However, when stratifying hypothermia in three levels, cats with ASA PS ≥III had a 76% reduction in the risk of developing anesthesia-related slight hypothermia compared to cats with ASA PS <III (RR = 0.24; 95% CI = 0.14–0.42; *P* < 0.0001). In addition, cats with ASA PS ≥III had 1.93 times and 2.37 times the risk of developing moderate (RR = 1.93; 95% CI = 1.24–3.00; *P* = 0.0036) and severe hypothermia (RR = 2.37; 95% CI = 1.51–3.73; *P* = 0.0002) than cats with ASA PS <III.

The analysis of the results of the studies (27, 28) combined found that dogs and cats ASA PS ≥III had 2.34 times the risk of developing severe hypothermia compared to patients with ASA PS <III (95% CI = 1.82–3.01; *P* < 0.001). No significant inconsistency was found between the results of the studies (*Q* = 0.006; *df* = 1; *P* < 0.9391; *I*^2^ = 0%).

The findings of Trim and Braun (36) indicated that the risk of anesthesia-related hypotension was not significantly different between pigs with ASA PS ≥III and pigs with ASA PS <III (RR = 1.27; 95% CI = 0.54–2.96; *P* = 0.5864).

## Discussion

This meta-analysis shows that for dogs, cats and rabbits with ASA PS ≥III the risk of anesthesia-related mortality up to 24 h (dogs) and up to 72 h (cats and rabbits) after anesthesia is higher than for dogs, cats and rabbits with ASA PS <III. In dogs, this increased risk is not consistent between the studies when the period of follow up is longer than 24 h. In addition, there was also evidence found to support that dogs and cats with ASA PS ≥III have an increased risk of developing severe hypothermia associated with anesthesia.

The present study indicates that part of the anesthesia-related deaths actually occurs after anesthesia and that, therefore, more attention should be given to this longer post-anesthetic period. Originally, the ASA PS classification was created in order to analyze data statistically and not to calculate operational risk (1). It was believed that the only cause of anesthetic mortality that could be correlated with the physical status of the patient was drug overdose (43) and human error (44). The increased risk of death during anesthesia could be attributed to the fact that animals with an ASA PS ≥III could not tolerate many anesthetic drugs due to their impaired functional organ systems. They could not compensate the cardiopulmonary alterations induced by the anesthetic drugs and, therefore, would be more likely to die during anesthesia. Sick animals could tolerate only a limited range of drugs, since the severe systemic disease could impair organ function that could not compensate for the hemodynamic alterations induced by the anesthetic drugs and death would be more likely to occur during anesthesia (24). In addition, these findings could suggest that stabilization of the patients prior to anesthesia in order to decrease the ASA PS category of the animal may be useful to decrease the risk of death. The surprising finding in the present study was that the increased risk of anesthesia-related death of patients with an ASA PS ≥III is significant during anesthesia until up to 24 h after the end of anesthesia in dogs and up to 72 h after the end of anesthesia in cats and rabbits. It suggests that death apparently does not occur only during anesthesia and, therefore, cannot necessarily be directly related to the use of certain anesthetic drugs. Other factors must be playing a role, of which the most likely one is progression of the underlying disease. Stress due to the disease, pain, and unfamiliar surroundings, can lead to anorexia, reduced gut motility, gastric ulceration, and immunosuppression, which could also contribute to the postanesthetic death. Other potential reasons could explain the higher risk of anesthesia-related death up to 24 h and up to 72 h after the end of anesthesia. Differences between studies in regards to the definition of anesthesia-related death, inclusion of sedated patients, and exclusion of those euthanized, and to the subjectivity of the ASA PS classification could have influenced these results. These features could also explain the significant inconsistency between the results of the studies assessing death after 24 h of the end of anesthesia in dogs.

The variation in the definition of anesthesia-related death among studies could have influenced the mortality rate in each ASA PS class. It could be difficult to distinguish the cases of mortality associated with anesthesia from those associated with the disease of the patient. In addition, terms as perioperative, postoperative, and perianeshetic death were often confused and used interchangeably throughout the studies with anesthesia-related risk of death without clear reference to their possible differences in meaning. Bille et al. (26, 30) and Clarke and Hall (19) included all deaths from medical or surgical complications and no attempt was made to classify the cause of death. In some studies, it was not clear whether ASA-associated risk of perioperative death could be interpreted as anesthesia-related. In the attempt to overcome this limitation, anesthesia-related death was defined as that where anesthesia could not be excluded as a potential cause, instead of only those where it was possible to ensure its association. In the studies of Itami et al. (33), Gil and Redondo (29), and Redondo et al. (28), it was possible to infer from the description of the causes of death, that they were associated with anesthesia or anesthesia could not be excluded as a potential cause of death. The studies of Robinson et al. (31) and Garcia de Carellan Mateo et al. (32) were analyzed separately because all deaths were included in the analysis (not only those anesthesia-related), and they were assessed in a specific population of dogs undergoing thoracic surgery and cats undergoing ureteral surgery, respectively. In rabbits, both studies of Lee et al. (35) and Brodbelt et al. (24) defined anesthesia-related death as any death that could not be explained totally by pre-existing medical or surgical complications, indicating that ASA-associated risk of perianesthetic death could be interpreted as anesthesia-related in this species.

In addition to the definition of anesthesia-related death, other potential explanations for differences among findings of the studies could be that two of them included sedated animals and six studies excluded deaths due to euthanasia from the analysis. All studies in rabbits included sedated animals (24, 35), indicating that rabbits with an ASA PS ≥III are at an increased risk of death associated not only with anesthesia, but also with sedation, compared with rabbits ASA PS <III. In dogs and cats, there was only one study (24) that included sedated animals, which could have had a minor impact in the differences among the findings included in the present analysis. In addition, the exclusion of euthanized animals could be associated with differences in the findings of the studies whenever anesthesia contributed to the negative outcome. However, all studies provided the reason for euthanasia and they did not seem to be associated with anesthesia.

The subjectivity of the ASA PS classification could have influenced the findings of the studies included in the present analysis. This subjectivity could be attributed to the vague definition of the ASA PS classes, especially before examples were published in the version of the ASA PS classification from 2014 (9). For instance, a healthy obese patient was cited as an example of an ASA PS II patient, while a morbid obese patient was cited as an example of an ASA PS III patient. However, Brodsky and Ingrande (45) stated that the physical status of a patient could not be based on his/her body mass index and that the obese population was heterogeneous. They specified that the presence of pathologies was independent of the bodyweight of the patient, and that it was the presence of fat infiltration that increases the risk of organic failure.

The subjectivity could lead to a high inter-observer and maybe intra-observer variability. In human medicine, some studies demonstrated a high inter-observer variability associated with pregnancy (46), smoking, the nature of surgery, airway complications, and acute injuries (47). The variability of the ASA PS scores was not correlated with the gender, age, expertise, working environment, or any demographic variable, and no difference between scores assigned by different groups of anesthetists was observed (47). In veterinary anesthesia, McMillan and Brearley (7) found a moderate variability among ASA PS scores given for 16 theoretical cases of small animals with different physical and pathological status by 144 anesthetists (specialist veterinarians, residents, interns, generalists and nurses). When studying real and non-hypothetical small animal cases in a university study, Mair and Wise (48) found homogeneity between ASA PS scores assigned by anesthetists and veterinary students. However, the inter-observer variability increased with the severity of the cases. In the present study, the Cochran's *Q* was calculated to verify the consistency of the findings of the studies in terms of whether they had the same outcome or not. The potential differences that could have contributed to the deviations remained unclear. In addition, the Newcastle-Ottawa scale was used to assess the quality of non-randomized studies included in the analysis and, therefore, the risk of bias. Finally, the ASA PS classes were grouped in I-II vs. III-V, which was described to improve the homogeneity of the responses in pediatric (49) and veterinary anesthesia (7).

The fact that the ASA PS score is a number does not make it an objective tool and, therefore, improvements in the classification were proposed to reduce inter/intra-observer variability. Some authors proposed the addition of a class of patients with moderate systemic disease between ASA PS II and ASA PS III (50). The lack of option for moderate systemic disease allowed the use of the ASA PS III as a cut-off to distinguish healthy from sick patients as previously described (19, 24), and to reach a binary answer to whether ASA PS was effective or not to identify patients at a greater risk of death or a specific complication. The simplification from a 5-point scale (ASA I-V) into a merged 2-point scale (ASA <III and ASA≥III), despite necessary to run the meta-analysis, could have resulted in some information loss.

It is debatable whether a patient with an ASA PS ≥III could also be associated with an increased risk of outcomes other than death. In human medicine, patients ASA PS class III-V had an increased cost of hospitalization (51). Accordingly, in veterinary medicine, a study with 235 dogs undergoing general anesthesia indicated that the ASA PS status was the only factor associated with the duration of ICU care (the higher ASA PS, the longer ICU stay), which, in turn, was a feature associated with an increase in the cost of hospitalization (34). In addition, the ASA PS classification could identify an increased frequency and severity of perioperative complications in human patients (52), dogs and cats (21, 22); a long ICU stay in dogs (34), and a poor recovery quality from anesthesia in horses (37, 38). Dogs ASA III, IV and V, were 3.4, 7.1, and 18.8 times, respectively, more likely to develop severe perianesthetic complications than those ASA I-II (21). Cats having an ASA status of III-V were nearly 4 times as likely to develop severe perianesthetic complications, such as cardiopulmonary arrest (22). In the present study, the risk of severe hypothermia in dogs and cats, and hypotension in pigs were anesthesia-related, but only the risk of hypothermia was associated with the preoperative ASA PS. The lack of association between the risk of hypotension associated with anesthesia and ASA PS in pigs may be associated with the small number of pigs included in the analysis (*n* = 27) (36). However, prospective studies with a large population would be necessary to confirm whether this is a true effect or type II error.

The search for evidence on whether the ASA PS classification can be recommended in veterinary anesthesia is a challenging task. The differences among studies (i.e., the length of follow up, definitions, inclusion and exclusion criteria, and subjectivity of the classification system) could have influenced the final analysis. However, some of these features cannot be controlled when assessing the risk of anesthesia-related death in patients with a high ASA PS. Indeed, randomized controlled trials, which presence greatly increases the quality of evidence (53), are not feasible. All prospective studies included in the present review were clinical observational cohort studies, which were not randomized and blinded, and did not control for mortality. In a clinical setting, patients were naturally randomized and mortality was not under control of the researcher. In addition, an independent blind assessment by assigning an ASA PS score without knowing whether the patient had a systemic disease is not possible. Usually cohort studies are not associated with a high quality of evidence because although they can show an association between an intervention and an outcome, they cannot prove a cause-effect relationship. However, it was never expected that an inadequate ASA PS score assigned preoperatively would cause anesthesia-related death. In order to answer our initial question, it was enough to know whether there was an association between the ASA PS score and the outcome.

Veterinary practitioners have obligations to inform owners of the potential foreseeable and serious risks their animal might be subjected to during a surgery. This review found evidence that dogs, cats, and rabbits with an ASA PS ≥III had an increased risk of anesthesia-related death and development of severe intraoperative hypothermia compared with those with an ASA PS <III. Nevertheless, the classification still needs to be refined to decrease inter and intra-raters variability. The outcome of anesthesia depends sometimes on other factors than the ASA PS status.

## Author contributions

KP contributed to the conception of the work, data acquisition and interpretation, drafted and revised the work, and approved the final version. KI contributed to the data acquisition and interpretation, drafted and revised the work, and approved the final version.

### Conflict of interest statement

The authors declare that the research was conducted in the absence of any commercial or financial relationships that could be construed as a potential conflict of interest.
